# The Role of Telomeres in Human Disease

**DOI:** 10.1146/annurev-genom-010422-091101

**Published:** 2022-06-24

**Authors:** Mary Armanios

**Affiliations:** Departments of Oncology, Genetic Medicine, Pathology, and Molecular Biology and Genetics; Telomere Center at Johns Hopkins; and Sidney Kimmel Comprehensive Cancer Center, Johns Hopkins University School of Medicine, Baltimore, Maryland, USA

**Keywords:** telomerase, aging, pulmonary fibrosis, clonal hematopoiesis, genome instability, cancer

## Abstract

Telomere biology was first studied in maize, ciliates, yeast, and mice, and in recent decades, it has informed understanding of common disease mechanisms with broad implications for patient care. Short telomere syndromes are the most prevalent premature aging disorders, with prominent phenotypes affecting the lung and hematopoietic system. Less understood are a newly recognized group of cancer-prone syndromes that are associated with mutations that lengthen telomeres. A large body of new data from Mendelian genetics and epidemiology now provides an opportunity to reconsider paradigms related to the role of telomeres in human aging and cancer, and in some cases, the findings diverge from what was interpreted from model systems. For example, short telomeres have been considered potent drivers of genome instability, but age-associated solid tumors are rare in individuals with short telomere syndromes, and T cell immunodeficiency explains their spectrum. More commonly, short telomeres promote clonal hematopoiesis, including somatic reversion, providing a new leukemogenesis paradigm that is independent of genome instability. Long telomeres, on the other hand, which extend the cellular life span in vitro, are now appreciated to be the most common shared germline risk factor for cancer in population studies. Through this contemporary lens, I revisit here the role of telomeres in human aging, focusing on how short and long telomeres drive cancer evolution but through distinct mechanisms.

## INTRODUCTION

Telomeres and telomerase were studied in simple model organisms long before any connection to human genetic disease was recognized (27, 28, 61). I have previously highlighted how these fundamentals have informed clinical paradigms for the diagnosis and treatment of patients with several conditions (14, 16). Here, I consider how contemporary clinical and human genetic observations uncover novel aspects of telomere biology that could not be readily inferred from cell-based systems or model organisms alone. In the first part, I review the role of short telomeres in human aging, focusing on how the evidence points to a prominent role that is largely limited to lung and hematologic disease. The short telomere syndromes are cancer-prone disorders, and I discuss how the recent discoveries point to immune and hematopoietic vulnerabilities as an underlying mechanism. Finally, I review the emerging body of evidence linking long telomeres to cancer risk, both in Mendelian syndromes and in the general human population, and outline how the latter findings uncover new fundamental biology that could be derived only from human genetics and within clinical contexts.

## TELOMERES AND TELOMERASE

Telomeres define the ends of linear chromosomes and are composed of tandem TTAGGG repeats bound by specialized proteins. They preserve genomic integrity by preventing chromosome fusion. Telomere length (TL) is heterogeneous within cells but generally shows a Gaussian distribution. The average length of the 92 telomeres in human leukocytes is a heritable trait (31); it is influenced by the parental TL as well as sequence variants (both rare and common) in telomere maintenance genes (17, 24, 46). TL shows a tight distribution across human populations, with reproducible upper and lower boundaries that range from 8 to 13 kb in leukocytes from newborns and thereafter shorten with age, normally at a constant rate (6, 23).

TL shortens with each cell division because chromosome ends cannot be fully copied during DNA replication (62). When a single or small group of telomeres reach a critical threshold, possibly around 100 base pairs, they signal a DNA damage response that provokes cells to undergo apoptosis or a permanent cell cycle arrest known as senescence (84, references in 18). TL predicts the onset of the Hayflick limit—the finite number of times that primary cultured cells can divide before reaching senescence (10, 47). As discussed below, while longer telomeres extend cellular longevity in vitro, they also increase the risk of cancer in the human population (66).

Telomerase is a specialized polymerase that evolved as the preferred mechanism for de novo telomere synthesis in nearly all organisms with linear chromosomes (27, 43). Telomerase has two essential components: a highly conserved reverse transcriptase, TERT, and a specialized telomerase RNA, TR, which provides the template for telomere repeat addition by TERT (44, 63, 68). A group of specialized proteins, known as shelterin, binds telomere DNA and functions to regulate telomerase recruitment and enzyme processivity (69). Shelterin components also protect chromosome ends from their recognition as double-strand breaks and from nuclease degradation (35).

Telomere elongation is highly orchestrated; it relies on intricate pathways involving RNA biogenesis, subcellular localization, and enzyme assembly along with coordinated recruitment to chromosome ends during S-phase. Recent studies have identified deleterious mutations in genes within and beyond those encoding the telomerase core components in Mendelian disease. Mutations in these components, which include shelterin as well as ones involved in RNA biogenesis, cause Mendelian syndromes associated with both short and long telomere extremes (39, 45, 59, 89, 96). Loci encompassing these same genes as well as others have also been identified in genome-wide association studies; they can similarly influence TL bidirectionally and influence disease risk, albeit with a smaller effect size (32, 66, 93).

### Short Telomere Syndromes Show Autosomal Dominant Inheritance

**Figure 1:**
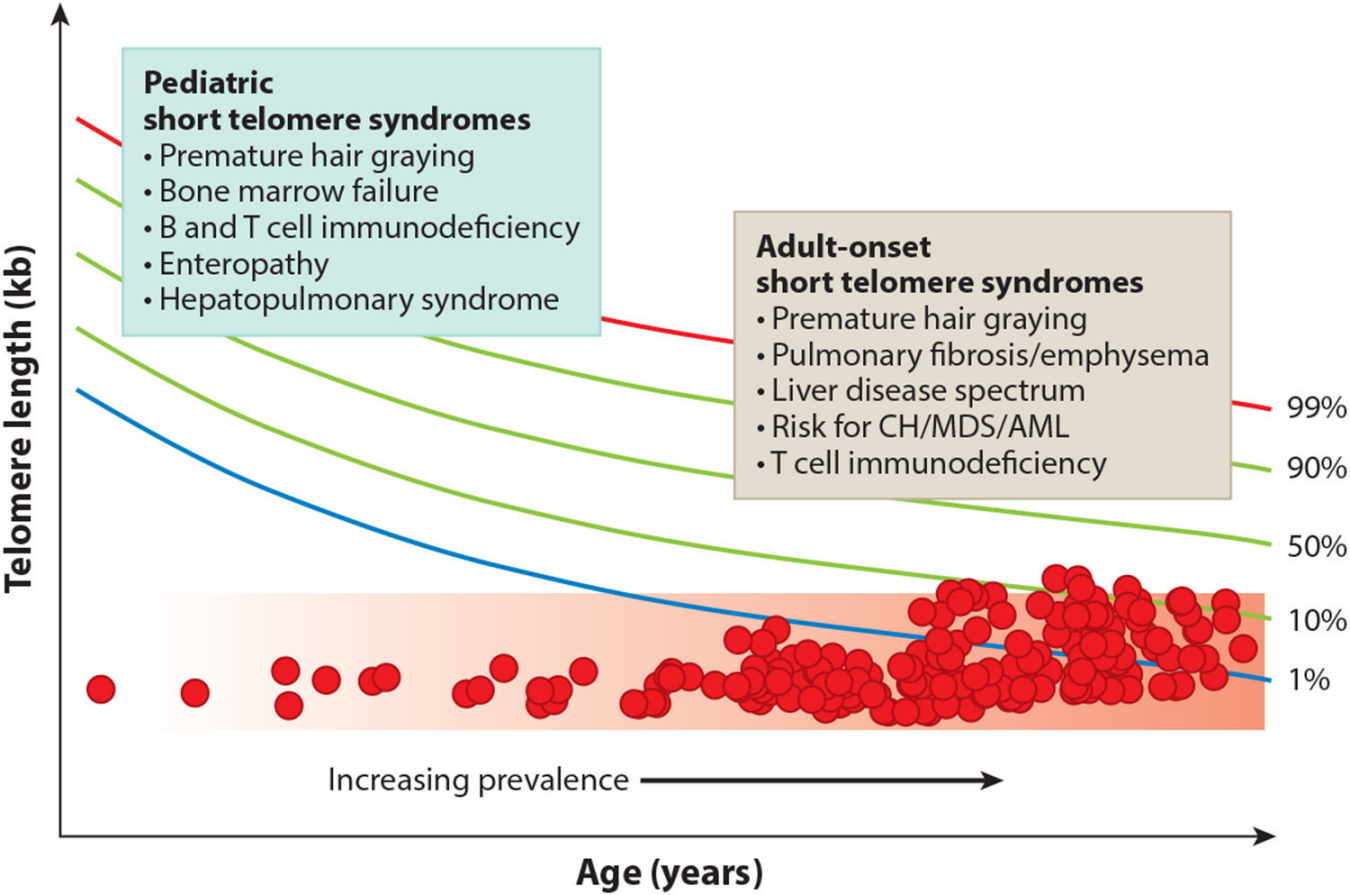
Distinct pediatric and adult manifestations of short telomere syndromes and their increasing prevalence with age. Short telomere syndromes show a phenotype continuum that is determined by the severity of the telomere length defect relative to age. The colored lines indicate percentiles for telomere length, and the two boxes summarize the common presentations at the extremes of age. Each red circle represents a hypothetical short telomere syndrome patient. The red shaded region indicates the threshold where short telomere length is sufficient to provoke telomere-mediated disease. Abbreviations: AML, acute myeloid leukemia; CH, clonal hematopoiesis; MDS, myelodysplastic syndrome. Telomere length nomogram adapted from Reference 6.

**Figure 2:**
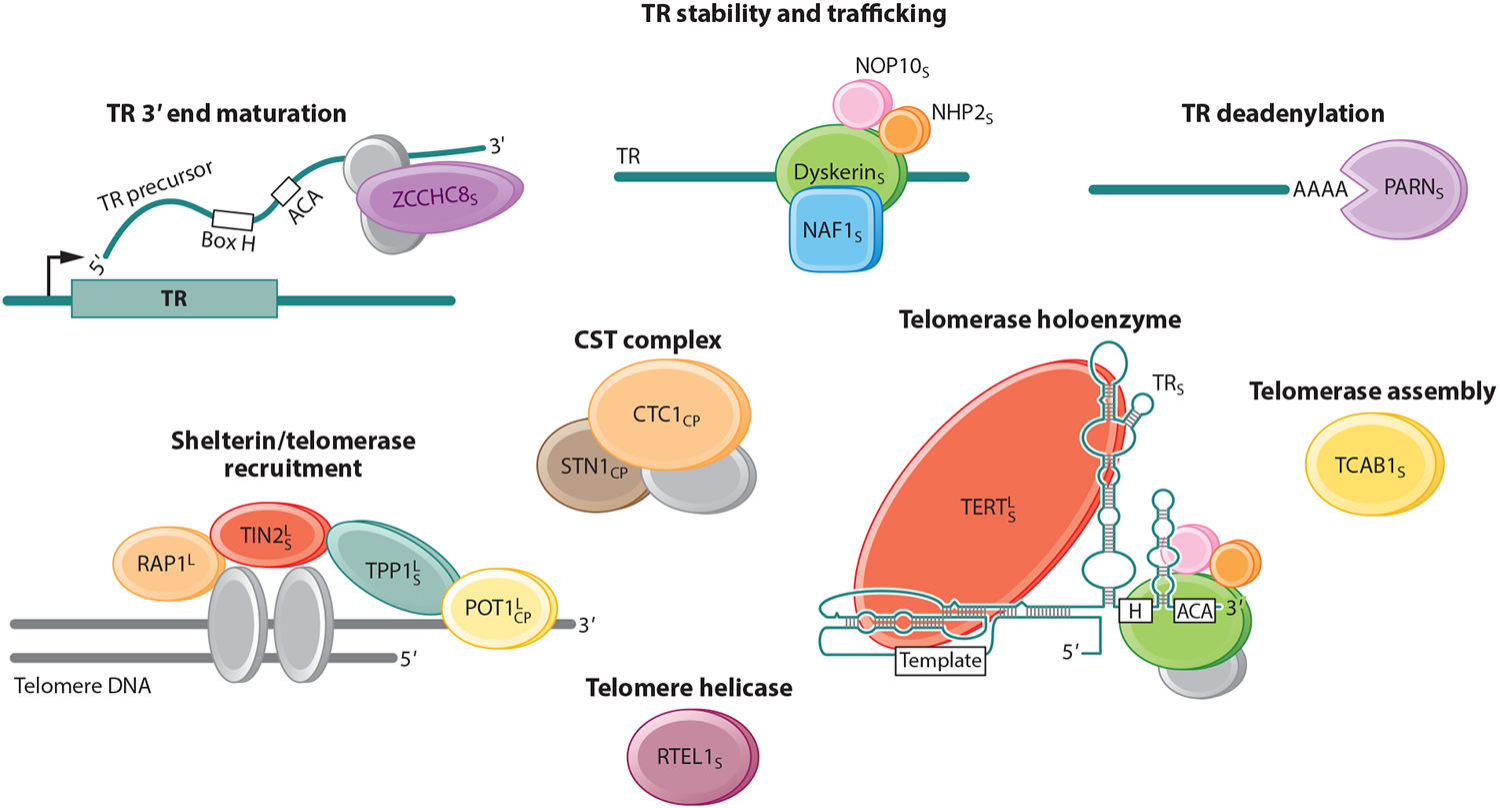
Telomere and telomerase components reported to be mutated in Mendelian telomere syndromes, grouped by their function when known. The proteins encoded by the 12 short telomere syndrome genes are indicated by a subscript S. Proteins encoded by Coats plus syndrome genes are indicated by a subscript CP; note that *POT1* mutations have been identified in only one patient, in the absence of segregation studies. Proteins encoded by genes identified as mutated in cancer-prone families based on their hypothesized function in telomere lengthening are indicated by a superscript L. RAP1 is encoded by *TERF2IP*, and TPP1 is encoded by *TPP1*, sometimes referred to as *ACD*. Figure adapted with permission from Reference 100.

The best-studied telomere disorders are the short telomere syndromes (18). They have a variable spectrum of severity that is determined by the extent of the short telomere defect (Figure 1). Mutations in 12 telomerase and other telomere maintenance genes explain approximately 60–80% of the inheritance in patients with Mendelian short telomere syndromes (Figure 2). Ninety percent of individuals with short telomere syndromes are diagnosed in adulthood, and they usually have a common age-related disease known as idiopathic pulmonary fibrosis (1). Short telomere syndromes are archetypal premature aging syndromes in that they recapitulate, albeit at an extreme, a process (telomere shortening) that occurs universally with aging. In half of the cases, heterozygous mutations in *TERT* are the culprit, and these autosomal dominant–inherited mutations show genetic anticipation in families (an earlier and more severe onset of disease across generations) (4, 20, 72). The genetic anticipation is mediated by the short TL, which is inherited along with the deleterious mutation (20, 46, 99).

**Figure 3:**
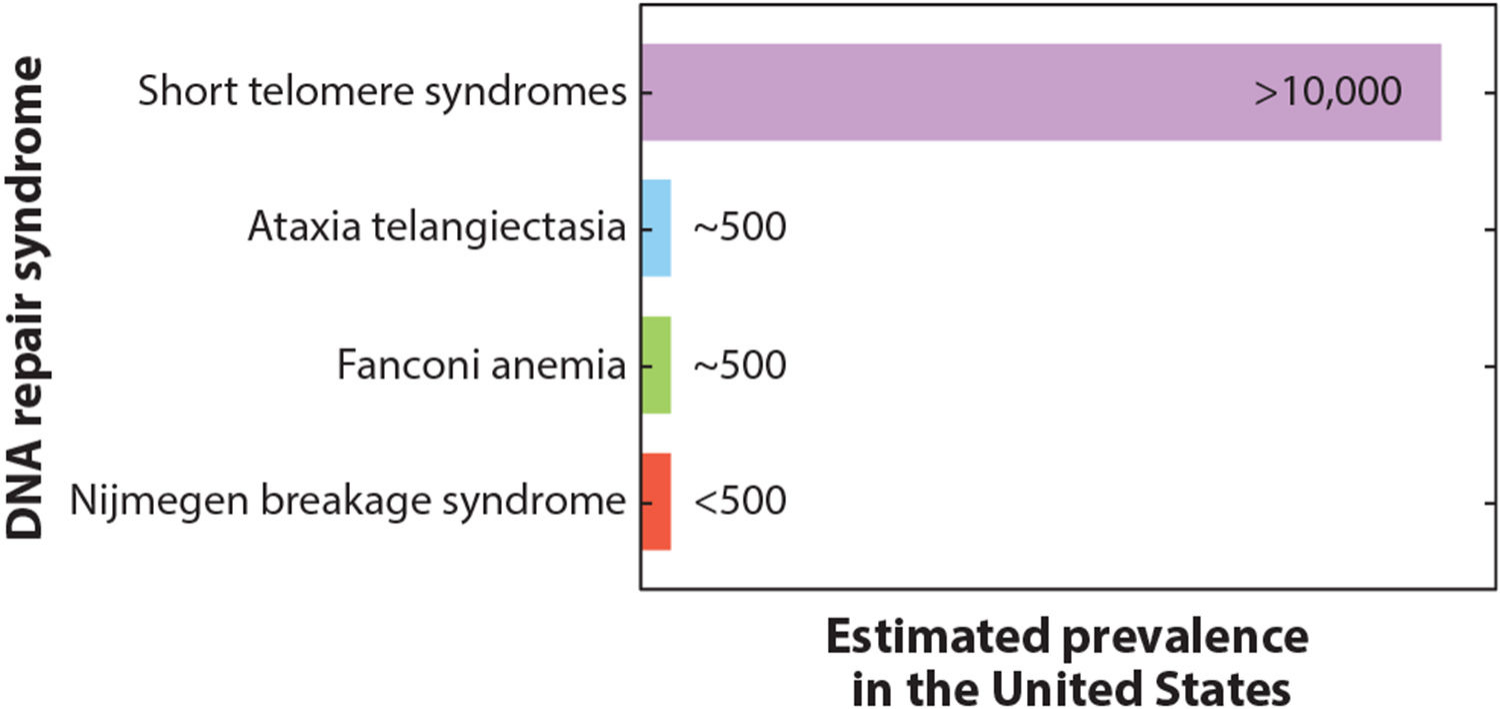
Estimated prevalence of short telomere syndromes relative to other classic DNA repair syndromes. The estimate for short telomere syndromes is based on the prevalence of short telomere mutations in idiopathic pulmonary fibrosis. Estimates for the other syndromes are based on the prevalence of rare pathogenic mutations in the respective genes.

Their autosomal dominant mode of inheritance and propensity for adult onset distinguish the short telomere syndromes from nearly all other classic DNA repair syndromes, including ataxia telangiectasia, Fanconi anemia, and Nijmegen breakage syndrome, which are autosomal recessive disorders usually diagnosed in childhood (94). The large proportion of patients with pulmonary fibrosis who also carry mutant telomerase genes also makes the short telomere syndromes the most prevalent of the premature aging disorders (Figure 3). Table 1 lists the mutant genes in Mendelian telomere syndromes and their modes of inheritance; the list includes the 12 genes identified to date as a cause of short telomere syndromes and the 3 genes that have been linked to the telomere-related autosomal recessive Coats plus phenotype (see below) (12, 20, 25, 33, 39, 45, 49, 59, 80, 86, 89, 91, 96-98, 101, 106). Figure 2 depicts the role of each of the genes in telomere maintenance where it is known.

### Multiple Constraints Prevent Excessive Telomere Elongation

The sensitivity of telomere maintenance to haploinsufficiency for telomerase and other genes reflects one fundamental aspect of telomere biology—that telomere synthesis has layered constraints on telomerase abundance and recruitment that are readily disturbed by heterozygous loss-of-function mutations. At steady state, TERT and TR are estimated to be present at approximately 20 molecules per cell, far fewer than all 92 telomeres that shorten during cell division (105). Underlying this disparity are multiple restraints on *TERT* transcription (60). Additionally, for TR, several components that regulate its biogenesis are also low in abundance (39, 89). As one illustration of the dosage sensitivity of TR to its biogenesis factors, heterozygous mutations in *ZCCHC8*, which encodes a protein involved in the maturation of the 3′ ends of hundreds of RNA polymerase II–transcribed RNAs, cause TR insufficiency and autosomal dominant short telomere disease, but other RNA functions are spared (39). However, a severe neurodevelopmental disorder related to widespread RNA dysfunction arises with biallelic *ZCCHC8* mutations (39).

## THE SHORT TELOMERE PREMATURE AGING PHENOTYPE

### The Pediatric Short Telomere Syndrome Phenotype Affects Tissues of High Turnover

One feature that distinguishes the short telomere syndromes from other Mendelian disorders is that even though affected patients generally share a single molecular defect (short telomeres), the predominant clinical phenotypes may at first glance appear distinct (Figure 1). Children and young adults (who have the most severe short telomere defects) develop disease in high-turnover tissues such as the bone marrow, while adults generally develop disease in the lung and liver, both of which are slow-turnover tissues. The entire spectrum may appear within a single family, where ancestral generations develop pulmonary disease while their children develop bone marrow failure (72).

The pediatric short telomere manifestations were historically the first to be recognized (49, 67). Short telomeres in these patients limit stem cell replicative potential, manifesting as bone marrow failure, immunodeficiency, and in some cases enteropathy (6, 56, 100). In rare cases, presentations may be recognized as a complex, such as with Hoyeraal–Hreidarsson syndrome, which is associated with intrauterine growth restriction (41, 51, 52), or dyskeratosis congenita, which is associated with mucocutaneous abnormalities (36). Patients with severe telomere-shortening defects diagnosed in infancy may carry biallelic mutations in *TERT* or other autosomal recessive mutations (Table 2). The availability of genetic testing and clinical TL measurements have established these syndromic entities (Hoyeraal–Hreidarsson and dyskeratosis congenita) as being rare, making up less than 5% of short telomere syndrome cases (6). Mutations in telomerase and other telomere maintenance genes encompass the largest subset of children and young adults with nonsyndromic inherited bone marrow failure, explaining 10% of cases (6, 29, 57), and recognizing these patients clinically has significant implications for treatment (6).

### Lung Disease Is the Predominant Manifestation of Short Telomere Syndromes in Adults

Idiopathic pulmonary fibrosis, an age-related disease of lung scarring, is the primary adult-onset manifestation of short telomere syndromes and is usually diagnosed after the sixth decade. Along with related interstitial lung diseases, it underlies mortality in the majority of adults with short telomere syndromes (1). Idiopathic pulmonary fibrosis has an estimated prevalence of 100,000 individuals in the United States alone (1), and a sizable subset of patients carry deleterious variants in telomere maintenance genes (Figure 3). For example, approximately one-third of familial cases of pulmonary fibrosis (20% of idiopathic pulmonary fibrosis cases are familial) carry one of eight telomere-shortening mutations (7, 8, 19, 33, 39, 40, 71, 89, 91, 95) (Table 1). And up to one-fourth of individuals with idiopathic pulmonary fibrosis undergoing lung transplantation (the only life-extending treatment for this disease) carry a mutation in a telomere maintenance gene (9).

One surprising observation that has derived from the human genetic studies has been that the lung is the most vulnerable organ in adults even though epithelial cell turnover is so slow, limited to a few times each year. The collective evidence points to a new paradigm for short telomere disease that is dependent not only on replication but on an inherited vulnerability to additional damage in a multistep model (reviewed in 1). To summarize briefly, the data point to lung-intrinsic and not circulating or inflammatory factors as the culprit (2, 5). Indeed, as in the bone marrow, short telomeres are sufficient to provoke stem cell senescence in the alveolar space, the region affected by scarring in pulmonary fibrosis (5). The stem cell senescence that is induced by short telomeres genetically is then exacerbated by endogenous and exogenous “second hits” that are acquired with aging to promote the fibrotic phenotype (2, 5). It is important to note that subtle phenotypes that resemble idiopathic pulmonary fibrosis are commonly seen radiographically and in autopsy studies of older individuals in the human population, suggesting that idiopathic pulmonary fibrosis is an aging phenotype in the lung (references in 1).

There are also sex-dependent factors, where women with short telomere syndromes—particularly those who have been exposed to cigarette smoke—are more prone to another lung aging phenotype known as emphysema (5, 90). The latter observations have highlighted a nuanced model where a gene–environment interaction (short telomeres and cigarette smoke) determines the predominant pulmonary phenotype (emphysema versus fibrosis), but this interaction is influenced by sex differences (5, 88, 90). The basis for these male–female differences, which emerge in the setting of cigarette smoke exposure, is not understood.

Adults with telomere-mediated pulmonary fibrosis–emphysema often also show some of the phenotypes seen in pediatric short telomere syndromes, although these may be at first milder (6, 76, 85). Bone marrow failure and immunodeficiency are often more pronounced after challenge with cytotoxic medications during lung transplantation and require individualized protocols (72, 83, 85, 100) (Figure 1). The hematologic malignancies myelodysplastic syndrome and acute myeloid leukemia (MDS/AML) affect 15% of adults after the age of 50 (83), and their clonal evolution is further discussed below. Ten percent of short telomere syndrome patients develop end-stage liver disease, with two relatively distinct manifestations among children and adults (30, 42, 70) (Figure 1). As TL measurement has been increasingly utilized in clinical settings (6), specialized protocols for affected patients are being increasingly adopted, and the evaluation of patients with lung disease and prior to lung transplantation is the most common indication for clinical TL testing in the United States (M. Armanios, unpublished findings).

### Coats Plus Syndrome Is a Rare Disease with Overlap with Short Telomere Phenotypes

One rare autosomal recessive disease (documented in fewer than 20 families) that has been linked to Mendelian telomere genetics is Coats plus syndrome. It is caused by biallelic mutations in *CTC1* and *STN1*, two genes that encode components of the CST complex, which plays a role in C-strand synthesis (12, 75, 86) (Figure 2; Table 1). One patient has also been identified with biallelic mutations in *POT1*, a single-strand telomere-binding protein (92). Although the exact pathophysiology remains incompletely understood, the Coats plus phenotype appears to encompass both TL-dependent (liver disease and bone marrow failure) and TL-independent (cerebral calcifications) pathologies (15, 34).

## THE ROLE OF TELOMERE SHORTENING IN HUMAN AGING BEYOND MENDELIAN DISEASE

**Figure 4:**
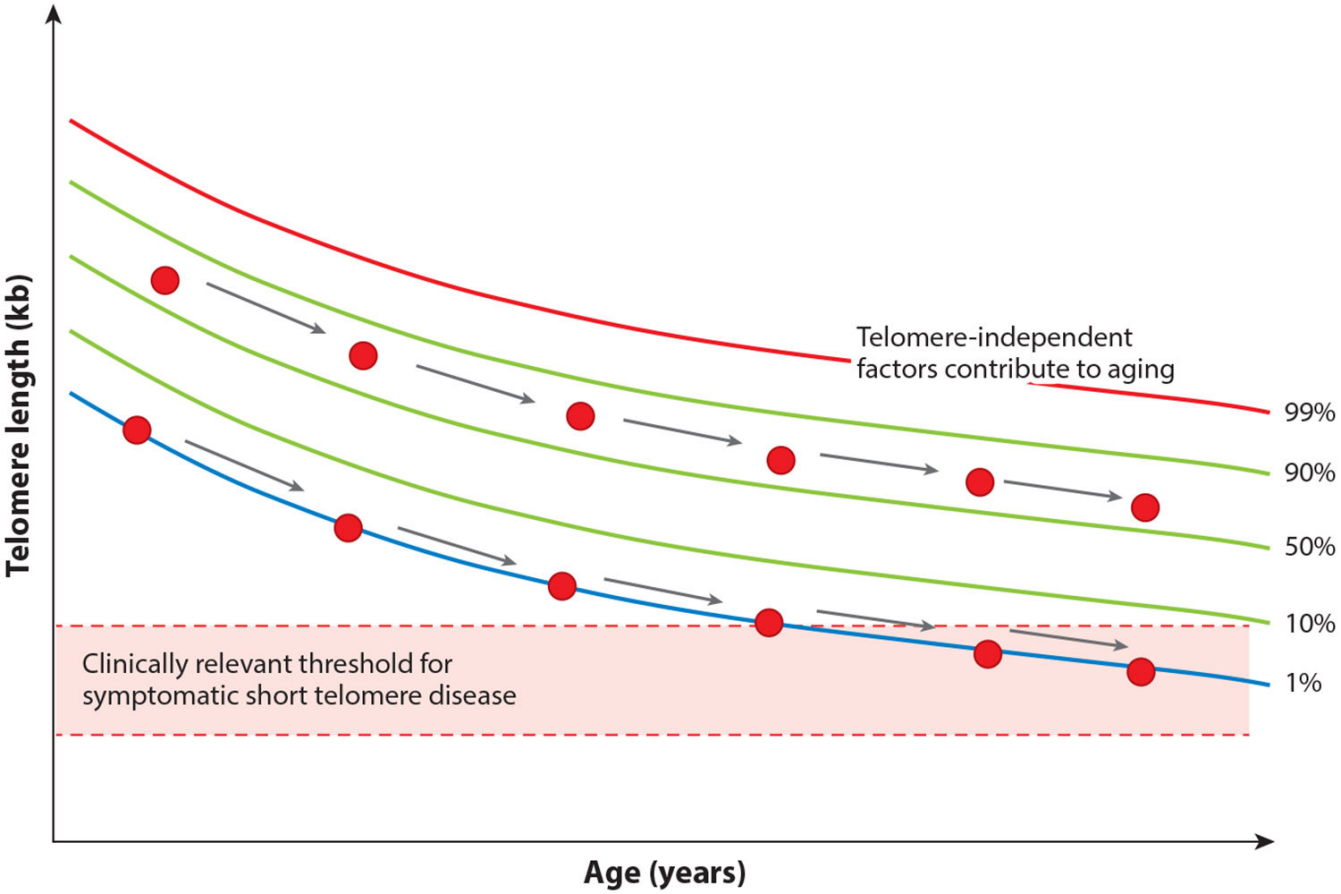
The short telomere threshold for disease, which is reached only in a subset of the human population. This scheme traces telomere shortening with age in two individuals (*red circles*), one with a telomere length near the median at birth and one with a telomere length in the lowest decile at birth. The threshold shown is based on data from short telomere syndrome patients (as also shown in Figure 1). Telomere length nomogram adapted from Reference 6.

The recent delineation of the short telomere syndrome phenotype provides a new opportunity to reconsider the role of telomere shortening broadly in human aging. For cells grown in vitro, telomere shortening is universally acquired, and TL determines the onset of the Hayflick limit. Several clinical observations, however, suggest that replicative senescence may not be reached in all humans, since some individuals who inherit longer telomeres have a buffer (the rate of telomere shortening is relatively constant with age) (Figure 4). In these cases, short telomeres are acquired, but the threshold for disease is not reached, and other factors would be expected to play a greater role in mediating disease risk (Figure 4).

There are three observations that support the idea that there is an absolute threshold for short telomere disease to manifest clinically. The first is that biologically, a threshold of short telomeres (estimated to be near 100 bases for a single or few telomeres in a cell) is required to provoke the DNA damage response. The second is the observation that there is also a clinically defined short TL threshold that is shared among short telomere syndrome patients where the risk of symptomatic disease increases (schematized in Figure 1 and described in more detail in Reference 6).

A third piece of evidence derives from the fact that the risk of lung disease is shared between individuals with inherited mutations in telomerase and those with short telomeres in the population (3). In one Mendelian randomization study of 400,000 individuals and 1 million controls that examined the effect of genetically determined TL on the risk for 82 diseases, including common cancers, individuals with the shortest telomeres had the highest risk for pulmonary fibrosis (32, 48). The effect was strong, with a 10-fold-higher risk, an effect that was unmatched relative to any other age-associated disease. These observations, along with the fact that the short telomere Mendelian phenotype is also notably absent for cardiovascular disease, neurodegenerative phenotypes, and the common malignancies, suggest that the role of telomere shortening in human aging is segmental and restricted (Table 2). In essence, even though telomere shortening may be one of the best-understood mechanisms of cellular aging, and is readily measurable, it does not explain all age-related disease risk, and it does so only at certain, now clinically defined, thresholds.

## TWO PATHWAYS TO CANCER DEVELOPMENT IN THE SHORT TELOMERE SYNDROMES

### Human Genetic Observations Diverge from Predicted Models Regarding Cancer Risk with Short Telomeres

Because age is the biggest risk factor for cancer, how short telomeres influence cancer risk has been a question of wide interest. Two lines of paradoxical evidence have emerged from cell-based and animal models. In the first set, in several mouse models of cancer driven by both oncogene and tumor suppressor mutations, animals with short telomeres repeatedly showed an improved cancer-free survival, consistent with a potent tumor suppressive effect (37, 73, 79, 104). In the one model where *Tp53* was deleted, however, mice with short telomeres developed more tumors, and these neoplasms showed hallmarks of genomic instability, including signatures of chromosome end-to-end fusions and breakage–fusion–bridge cycles (21). Cells with telomere dysfunction also normally undergo senescence, but when lacking *TP53*, they can bypass this checkpoint and show genome instability, including catastrophic events such as chromothripsis (65). In past decades, the animal and cell culture evidence has favored a dominant interpretation that emphasized short telomeres as a driver of genome instability and cancer progression with aging (22, 38, 64).

**Figure 5:**
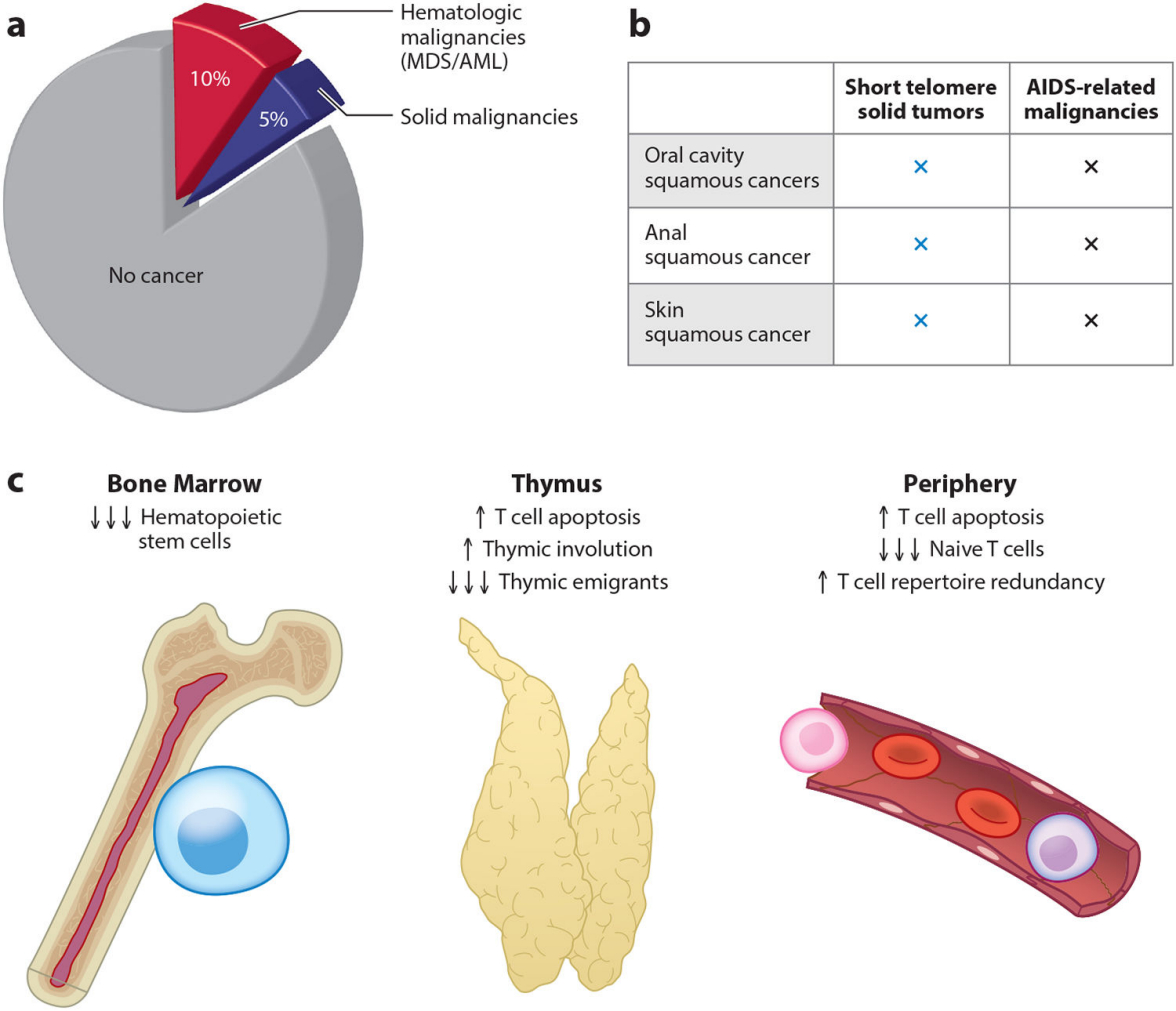
Cancer prevalence and association with T cell immunodeficiency in short telomere syndromes. (*a*) Pie chart showing a 15% cancer prevalence in short telomere syndromes, broken down into hematologic (MDS/AML) and solid malignancies. (*b*) Overlap in the solid tumor spectrum with malignancies associated with T cell immunodeficiency, such as in AIDS. (*c*) Mechanisms of T cell immunodeficiency in short telomere syndromes, which are compounded throughout T cell development in humans and mice. Factors include depleted hematopoietic progenitors in bone marrow, increased T cell apoptosis in the thymus during development, and increased T cell apoptosis among mature T cells. Abbreviations: AIDS, acquired immunodeficiency syndrome; AML, acute myeloid leukemia; MDS, myelodysplastic syndrome.

In the last few years, there has been an opportunity to test the consequences of short TL on human cancer in both Mendelian and epidemiologic contexts. One of these observations relates to the prevalence of cancer in the short telomere syndromes, which is higher relative to the population, but the lifetime risk is 15%, which is significantly lower than the risk in other classic cancer predisposition syndromes, such as Li–Fraumeni or Lynch syndrome (where it may reach 80% or higher) (11, 81, 83) (Figure 5a). The spectrum of malignancies is also narrow and distinct from the common age-related malignancies (i.e., it excludes lung, colon, and pancreatic cancer; Table 2). The most common short telomere malignancies are MDS and AML, which constitute two-thirds of short telomere syndrome malignancies (Figure 5a). The remaining cancers show squamous histology and are derived from skin and mucosal epithelium, with these solid tumors (i.e., nonhematologic) having an approximately 5% lifetime risk (81, 83) (Figure 5a). Analysis of the somatic landscape of these patient-derived tumors indicates that they do not show the hallmarks of genome instability seen in in vitro models (83; K.E. Schratz & M. Armanios, manuscript in preparation), pointing to other factors contributing to their evolution. The observation that individuals with short TL in the human population also have a relatively low risk of age-related malignancies (48) is consistent with these results, since MDS/AML and squamous cancers are relatively rare with aging (Table 2).

### T Cell Immune Surveillance Defects, Rather than Genome Instability, Explain the Short Telomere Solid Tumor Spectrum

**Figure 6:**
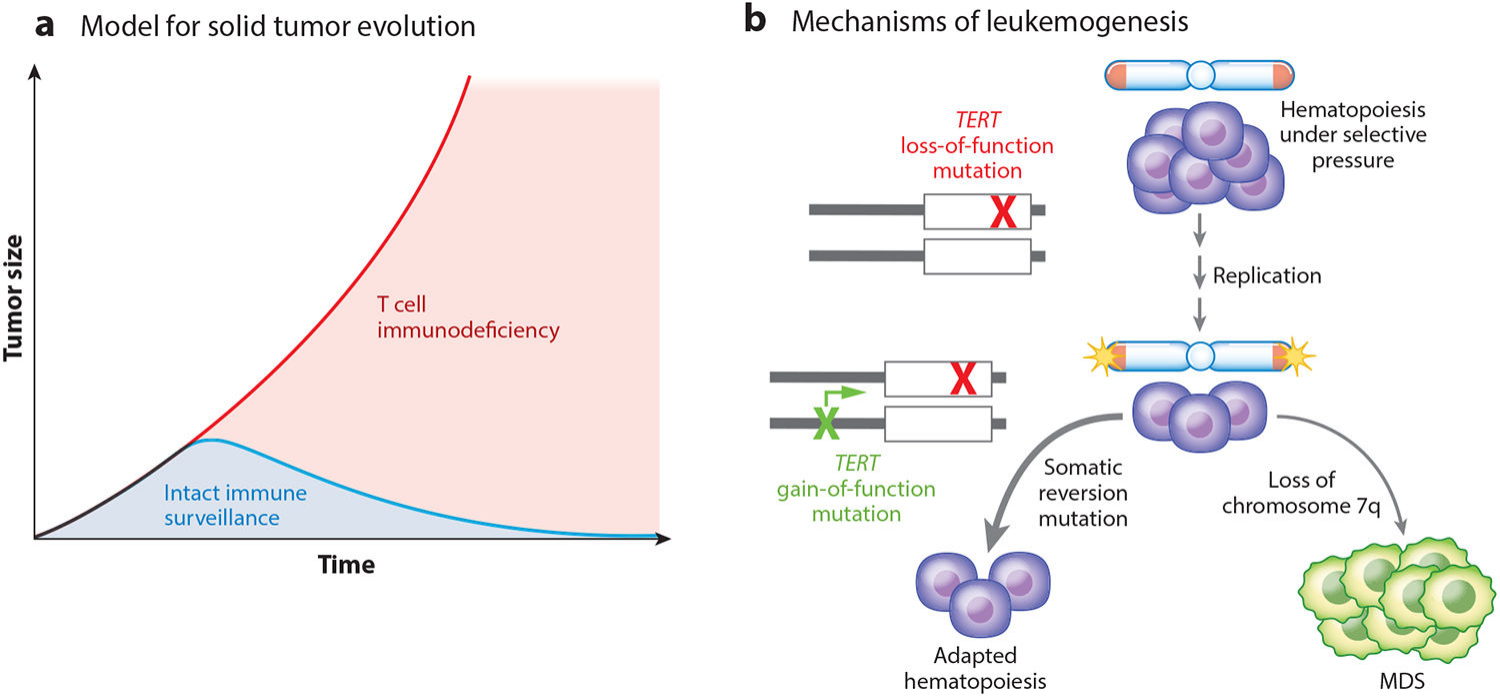
Models for short telomere cancer risk in solid organs and in the bone marrow based on recently generated data. (*a*) Model for solid tumor evolution in short telomere syndromes. In this hypothesized mechanism of carcinogenesis, T cell immunodeficiency promotes defective immune surveillance to promote cancer risk, especially in squamous cell compartments. (*b*) Mechanisms of leukemogenesis. Acquired somatic reversion in adults with short telomere syndromes at high allele frequency averts the telomere crisis that leads to MDS or leukemogenesis. The latter model is based on data showing a higher prevalence and mutual exclusivity of somatic reversion mutations with cytogenetic abnormalities (based on Reference 82). Abbreviation: MDS, myelodysplastic syndrome.

A closer examination of the short telomere syndrome solid cancer spectrum shows that it overlaps with cancers that arise in patients with T cell immunodeficiency, such as those with acquired immunodeficiency syndrome (AIDS) and those who have undergone solid organ transplantation (Figure 5b). Short telomeres have been extensively documented to limit every aspect of T cell development, both in patients with short telomere syndromes and in mouse models (Figure 5c). For example, the production of T cell precursors is limited by depleted hematopoietic stem cells, and even T cells that migrate to the thymus have a higher rate of attrition by apoptosis throughout their development (100). The cumulative effect is premature thymic involution, which in turn limits the export of circulating naive T cell pools (100). Mature circulating cells that reach the periphery, including memory T cells, are prone to apoptosis, and with age, this leads to their dropout, resulting in a restriction of the T cell repertoire diversity and contributing to a risk of reactivation of latent viruses (76, 100) (Figure 5c). These data point to the solid tumor–prone state in the short telomere syndromes as being distinct from the cancer-prone state normally associated with aging, and it is potentially explained by defects in T cell–mediated immune surveillance (Figure 6a).

### Short Telomere Length Promotes Adaptive Clonal Hematopoiesis

The most common malignancies are MDS and AML, with a nearly 100-fold-increased prevalence among short telomere syndrome patients, even though the overall penetrance is relatively low (lifetime risk of 10%) (83) (Figure 5a). The majority of patients develop MDS/AML as adults after the age of 50, consistent with age-dependent factors playing a role, but the age of onset of MDS/AML in short telomere syndrome patients is two decades earlier than in the general human population (83). Telomere genetics are relevant for understanding the germline susceptibility to MDS broadly since 3–5% of adult patients with MDS carry a pathogenic mutation in *TERT* (58, 77). These findings establish the short telomere pathway as one of the most (if not the most) common Mendelian causes of adult-onset MDS.

How does telomere shortening increase the risk of MDS/AML? Normally, the DNA damage response provoked by short telomeres limits the proliferative potential of hematopoietic stem cells and leads to bone marrow failure, as is the case in children with aplastic anemia (6, 72). Among adults, however, the predominant bone marrow failure phenotype is MDS/AML, a state that is associated with morphologic abnormalities of bone marrow progenitors as well as clonal mutations. MDS/AML arising in patients with short telomere syndromes lacks the hallmarks of genome instability, although patients acquire cytogenetic adaptations (such as loss of chromosome 7) that are shared with other inherited bone marrow failure syndromes (83). Notably, among short telomere patients who do not show MDS/AML, a premature onset of clonal hematopoiesis that is normally acquired with aging is also seen but occurs two decades earlier (83). These observations have raised the possibility that short telomeres may be one driver of age-related clonal hematopoiesis in the human population (54, 83), a question that remains to be definitively answered.

### Somatic Reversion May Protect Against the Evolution of Myeloid Malignancies

One conundrum in dissecting the cancer biology of short telomere–associated cancers is how they sustain their proliferation. A recent study examined this question, hypothesizing that somatic reversion mutations (within the same gene or indirectly through a compensatory mutation) are advantageous in the highly proliferative hematopoietic compartment and offset the germline defect (82). The results were surprising because the prevalence of these mutations was very low in MDS/AML patients. By contrast, one-third of adults without MDS/AML were found to carry a functional reversion mutation in a gene that was predicted to promote telomere lengthening (82). These somatic genetic reversion events were identical to telomere maintenance mechanisms seen in many cancers, but in this context, they seemed protective against MDS/AML risk (Figure 6b). The most common mutations fell in the *TERT* promoter canonical sites, which upregulate *TERT* transcription (50, 53), but they also included loss-of-function mutations in *POT1*, a negative regulator of telomere elongation (82) (Figure 6b). Highlighting their functional role, the somatic reversion mutations were in general mutually exclusive with cytogenetic abnormalities characteristic of telomere-mediated MDS/AML, such as monosomy 7 (82). These observations support a new model of leukemogenesis that is relevant and may be useful for clinically assessing the risk of MDS/AML development: Under the selective pressures of telomere shortening with age, in the highly replicative compartment of hematopoiesis, adaptive suppressor mutations arise, restoring homeostasis (Figure 6b). More rarely, maladaptive cytogenetic abnormalities promote MDS/AML evolution (Figure 6b).

## LONG TELOMERES AND CANCER RISK

### The Long Telomere Cancer Spectrum Encompasses the Common Malignancies Associated with Aging

In contrast to the observations supporting a low penetrance and narrow spectrum of cancer associated with short telomeres, recent evidence has somewhat unexpectedly supported a greater role for long telomeres in promoting age-related cancer risk. The data derive from observations of families with cancer predisposition as well as population studies (reviewed in 66). Mutations in five telomere-related genes (the *TERT* promoter and the shelterin genes *POT1, TPP1, TERF2IP*, and *TINF2*) have been linked to the risk of familial melanoma, glioma, and chronic lymphocytic leukemia, and they explain 1–10% of families with these cancers and show autosomal dominant inheritance (see references in 66) (Figure 2; Table 1). The mutations have a net effect of increasing telomerase dose, such as with the *TERT* promoter, or removing negative regulation of telomerase elongation, as is hypothesized currently for the shelterin mutations.

Evidence from epidemiologic studies aligns with these data, showing a similar effect of long telomeres, even in the absence of these Mendelian mutations, in predisposing to the same malignancies. By way of example, common polymorphisms near *TERT*, which are associated with longer telomeres, are the most common germline single-nucleotide variants associated with a wide variety of cancers, including melanoma and glioma (reviewed in 55, 66). Long TL itself, as directly inferred from polygenic risk scores, is also associated with an increased incidence of the common cancers associated with aging, including lung and ovarian cancers, melanoma, and glioma (48, 78; reviewed in 66). The current model is that long telomeres in these cases sustain the longevity of somatically acquired mutations with aging, which eventually allows for cancer evolution, as had been previously observed in long telomere mouse models (reviewed in 66). The overall data underscore the delicate balance between the pro-aging effects of short telomeres and their advantageous role in tumor suppression.

### The Contrast Between Short and Long Telomere Phenotypes

**Figure 7:**
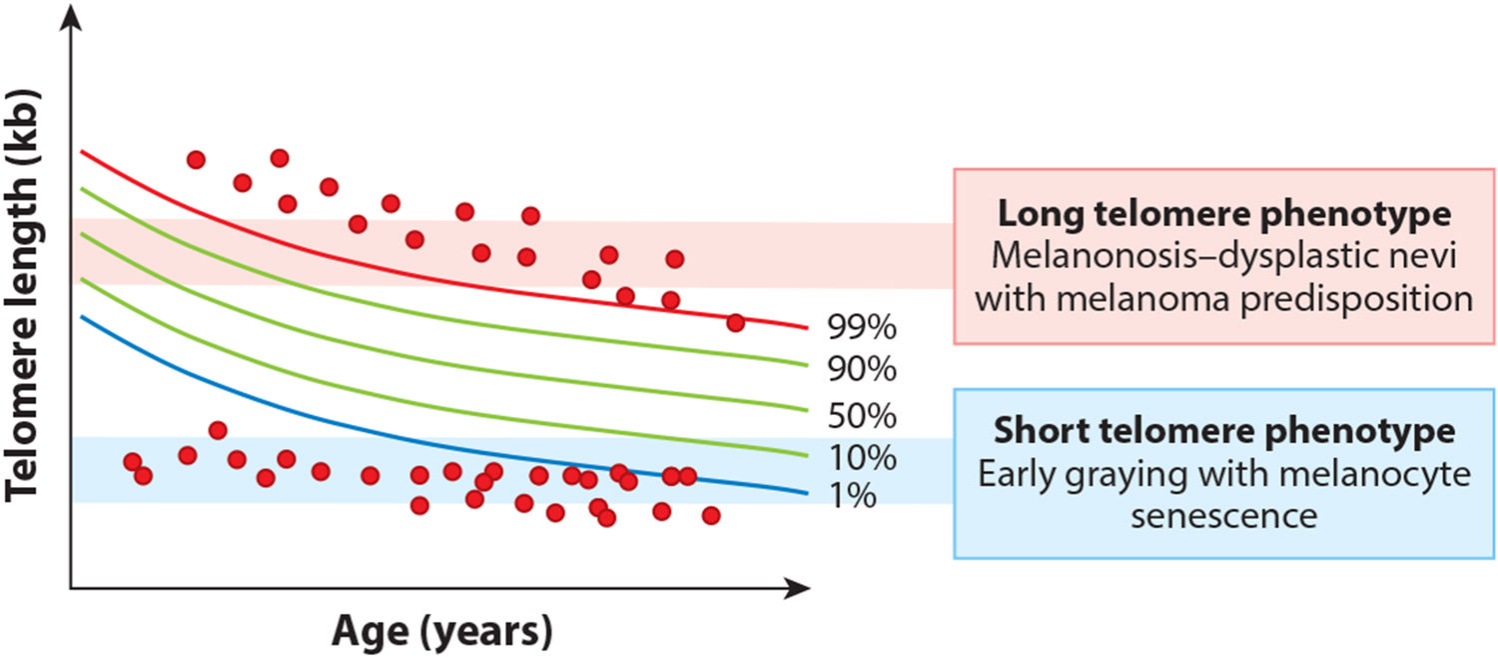
Schema showing hypothesized long and short telomere syndrome patients (*red circles*) with telomeres at extremes of the normal range. The melanoma phenotype in patients with long telomeres contrasts with the melanocyte loss seen in the premature graying phenotype of the short telomere syndromes, demonstrating how the short telomere aging phenotype is tumor suppressive at this extreme. Telomere length nomogram adapted from Reference 6.

Although the complete long telomere syndrome spectrum remains incompletely understood (87), there is an observable and notable contrast with short telomere phenotypes. Melanoma was the first cancer to be associated with telomere-lengthening mutations, and 4% of familial cases of melanoma harbor a mutation in a telomere-lengthening gene (66) (Table 1). The melanoma neoplastic phenotype contrasts with the premature hair graying seen in patients with short telomere syndromes, which is likely caused by melanocyte senescence (Figure 7). A similar contrast of phenotypes may also be present in T cells, where a higher incidence of T cell lymphomas manifests in patients with telomere-lengthening mutations, contrasting with the profound T cell immunodeficiency seen in the short telomere syndromes (100, 103). The long telomere syndrome cancer spectrum, if further elucidated, may uncover a novel cancer predisposition mechanism that is tied to extended cellular longevity and that is distinct from the known cancer-prone syndromes caused by mutant proto-oncogenes and tumor suppressors.

### Mutations in Three Genes Have Paradoxical Effects on Telomere Length

Another opportunity that emerges from the study of human genetics relates to the surprising implications for understanding fundamentals of TL regulation that are not inferred from in vitro systems alone. Different mutations in identical telomere genes appear to have bidirectional effects on TL. In three distinct examples (Figure 2; Table 1), mutations in the same gene, but in a distinct domain, mediate a short or long Mendelian telomere phenotype. The first of these to be identified were the gain-of-function germline *TERT* promoter mutations, which predispose to autosomal dominant melanoma in rare cases (50). They contrast with the loss-of-function short telomere mutations so commonly identified in the short telomere syndromes (20). For the other two proteins, the contrasts are more complex because the mutations may all be classifiable as having a loss-of-function effect even though they have bidirectional consequences for the TL. For the shelterin component TPP1 (encoded by *ACD*), nonsense mutations are hypothesized to remove a negative regulation on telomerase elongation, and these mutations have been implicated in familial melanoma (13). By contrast, mutations that fall in specific residues within the TEL patch, a domain required for TERT recruitment, cause a short telomere phenotype (45, 59). Similarly, deleterious frameshift mutations in *TINF2*, which were recently described in familial thyroid cancer (102), contrast with the recurrent hot-spot mutations that cause telomere shortening by interfering with telomerase recruitment or processivity (74). In these latter examples, the human genetic data paint a more nuanced view of TL regulation that remains to be fully characterized, with implications for large effects on disease risk.

## SUMMARY AND FUTURE OPPORTUNITIES

The discovery of telomeres and telomerase through elegant experiments in simple model organisms has provided a starting point for understanding fundamental mechanisms of cellular longevity. This knowledge has helped in dissecting the etiology, biology, and treatment of several common and previously considered idiopathic diseases. However, recent and ongoing human genetic and clinical observations now in turn raise questions regarding previously generated models as they relate to the role of telomeres in disease, including human aging and cancer. The unraveling of these clinical contexts, for both short and long telomere disease, opens new possibilities that have the potential to uncover a greater role for telomeres in human disease than has been previously appreciated, albeit in unexpected settings.

Some of the remaining questions point to potential new areas of research, such as how TL changes with age influence the evolution of clonal hematopoiesis beyond the Mendelian syndromes. This question is timely since variants near *TERT* are the most statistically significant inherited risk factor for clonal hematopoiesis (26). The idea that short telomeres limit T cell function in profound ways also holds promise for understanding the biology of squamous cell cancers, an understudied group of tumors. It may also provide predictive biomarkers for the efficacy of immune checkpoint inhibitors that are widely used in the treatment of squamous cancers. The association of long telomeres with cancer risk also promises new possibilities for patient care in paradigms related to cancer prevention, early detection, and treatment. The curiosity-driven discovery of telomeres and telomerase exemplifies how fundamental science can transform clinical paradigms. There is now a new opportunity to align these paradigms with contemporary clinical and genetic discoveries in new directions that also inform a deeper molecular understanding of the complexities of TL regulation.

## Figures and Tables

**Table 1:** Mutant genes in Mendelian telomere syndromes by function, along with their modes of inheritance

Mutant gene	Telomere shortening	Telomere lengthening	Mode of inheritance
**Telomerase core**			
*TERT*	X	X	AD, AR
*TR*	X		AD
**TR processing and biogenesis**			
*DKC1*	X		X linked, de novo
*ZCCHC8*	X		AD
*NAF1*	X		AD
*PARN*	X		AD, AR
*NOP10*	X		AR
*NHP2*	X		AR
**Telomere-binding proteins**			
*TPP1*	X	X	AR
*TINF2*	X	X	AD, de novo
*POT1* ^a^		X	AD
*TERF2IP/RAP1*		X	
**Other**			
*RTEL1*	X		AD, AR
*TCAB1*	X		AR
**Coats plus syndrome** ^b^			
*CTC1*	X/−		AR
*STN1*	?		AR

Abbreviations: AD, autosomal dominant; AR, autosomal recessive.

a *POT1* homozygous mutations have been limited to one patient with Coats plus syndrome.

b Published results vary as to whether patients with Coats plus syndrome have telomere length abnormalities.

**Table 2:** Prevalence of different disease phenotypes with age and in the short telomere syndromes

Phenotype	Prevalence with age	Prevalence in short telomere syndromes
Hair graying	•••••	•••••
Cardiovascular disease	•••••	
Cancer		
Lung	•••	
Colon	•••	
Breast	•••	
Pancreas	•••	
Prostate	•••	
Squamous skin/head and neck	•	••
Myelodysplastic syndrome	•	•••••
Neurodegenerative disease	•	
Pulmonary disease		
Idiopathic pulmonary fibrosis	•	•••••••••••
Emphysema	•••	•

The number of circles for each annotation refers to the estimated relative prevalence of this particular phenotype within each group and highlights the preponderance of lung disease and myelodysplastic syndromes among short telomere syndrome patients.
